# Functional Variants in MicroRNAs (rs895819, rs11614913 and rs2910164) Are Associated with Susceptibility and Clinicopathological Features in Mexican Patients with Colorectal Cancer

**DOI:** 10.34172/aim.2023.67

**Published:** 2023-08-01

**Authors:** Yuri Giovanna Vanessa Trujillo-Fernández, Carmen Yzabal-Barbedillo, Anilú Margarita Saucedo-Sarinaña, César de Jesús Tovar-Jácome, Miriam Yadira Godínez-Rodríguez, Patricio Barros-Núñez, Martha Patricia Gallegos-Arreola, Clara Ibet Juárez-Vázquez, Tomás Daniel Pineda-Razo, María Eugenia Marín-Contreras, Mónica Alejandra Rosales-Reynoso

**Affiliations:** ^1^Molecular Medicine Division, Centro de Investigación Biomédica de Occidente, Instituto Mexicano del Seguro Social (IMSS), Guadalajara, Jalisco, México; ^2^Doctorado en Genética Humana, Centro Universitario de Ciencias de la Salud, Universidad de Guadalajara, Jalisco, México; ^3^Department of Devices and Systems I, Facultad de Medicina. Decanato Ciencias de la Salud, Universidad Autónoma de Guadalajara (UAG). Zapopan, Jalisco, México; ^4^Genetic Division, Centro de Investigación Biomédica de Occidente, Instituto Mexicano del Seguro Social (IMSS), Guadalajara, Jalisco, México; ^5^Medical Oncology Service, Hospital de Especialidades, Instituto Mexicano del Seguro Social (IMSS), Guadalajara, Jalisco, México; ^6^Gastroenterology Service, Hospital de Especialidades, Instituto Mexicano del Seguro Social (IMSS), Guadalajara, Jalisco, México

**Keywords:** Cancer Risk, Colorectal cancer, *miR-146a*, *miR-196a2*, *miR-27a*, TNM stage, Tumor location

## Abstract

**Background:**

miRNAs are non-coding RNAs participating actively in the post-translational regulation of oncogenes, tumor suppressor, and DNA repair genes implicated in colorectal cancer (CRC). This study aims to examine the association of the variants *miR-27a* (rs895819 A>G), *miR-196a2* (rs11614913 T>G) and *miR-146a* (rs2910164 C>G) in Mexican CRC patients.

**Methods:**

DNA samples from 183 patients and 186 healthy Mexican subjects were analyzed. Variants were identified by polymerase chain reaction-restriction fragment length polymorphism (PCR-RFLP) methodology. Association was calculated by the odds ratio (OR) and adjusted by the Bonferroni test.

**Results:**

Patients carrying the G/G genotype of the rs895819 variant in the *miR-27a* gene showed an increased risk of CRC (19% vs 12%, *P*=0.013). A similar tendency was noticed for patients younger than 50 years carrying A/G (48% vs 41%, *P*=0.014). The A/G genotype in TNM stages I+II (55.7% vs 40.8%, *P*=0.011) and tumor location in the colon (69.5 vs 40.8%, *P*=0.001) were also increased. For the variant rs11614913 of the *miR-196a2* gene, carriers of the C/C genotype showed an increased risk of CRC (32% vs 22%, *P*=0.009). This genotype was more frequent in TNM stage III+IV (36.8% vs 22.5%, *P*=0.007) and the tumor had a more recurrent location in the rectum (31.6% vs 22.5%, *P*=0.013). The rs2910164 variant of the *miR-146a* gene was found to have no significant risk associations.

**Conclusion:**

Our results reveal that the rs895819 variant in *miR-27a* and rs11614913 in *miR-196a2* have a substantial impact on the development of CRC.

## Introduction

 Colorectal cancer (CRC) is the third cancer with the highest incidence and the second highest mortality rate in Mexico and worldwide.^1^ In 2021, its incidence reached a rate of 11.6 per 100 000 and its mortality was 5.4 per 100 000 inhabitants in Mexico.^2^ CRC is a complex and multifactorial disease whose complexity involves genetic, epigenetic, environmental and lifestyle factors.^2^ Previous studies show that microRNAs (miRNAs) play a key role in numerous biological processes such as tumorigenesis, proliferation, apoptosis, metastasis and multidrug resistance of cancers.^3^ miRNAs are short (18-25 nucleotides), non-coding and single-stranded sequences, functioning as negative regulators of gene expression.^4^ Single nucleotide polymorphisms (SNPs) in a number of miRNA genes result in several functional consequences, including cancer development, progression and metastasis.^5^

*miR-27a* localizes to 19p13.13 and is involved with susceptibility for CRC; multiple studies demonstrate that *miR-27a *is associated with various types of cancer^6-9^ including CRC.^6^ The rs895819 A > G variant is in the pre-miR-27a loop. The change of A to G may alter its secondary structure, with an aberrant expression of its target gene and affectation of the miRNA function, with cancer promotion or tumor suppression.^9,10^ Recently, a number of studies have identified the important role of this variant in gastric cancer,^11,12^ CRC,^6,7,12-14^ breast cancer^15^ and cervical cancer.^7^ In CRC, controversial results have been described for the rs895819 variant.^13-16^

 The *miR-196a2* is located on 12p13.13 and is actively involved in tumor invasion and metastasis.^17^ The most extensively investigated miRNA-associated SNP in cancer is rs11614913 C > T of *miR-196a2* (pre-miRNA region) and its association with breast,^18^ lung,^19^ gastric,^20^ esophageal,^21^ hepatocellular,^22^ colorectal,^16^ head and neck,^23^ prostate and hepatocellular cancer,^21^ cancer has been documented. The results observed for the association of rs11614913, and CRC, however, are inconclusive.^16^

*miR-146a* is located at 5q33.3 and is associated with CRC,^5^ prostate cancer,^17^ lung cancer^19^ and breast cancer.^9,15^ The rs2910164 C > G variant in pre-miRNA-146a leads to a change from a C:U pair to a G:U pair that affects the integrity and expression of mature *miR-146a *and its target genes. This variant is associated with CRC,^24^ digestive^25^ and hepatocellular cancer.^26^ However, results involving rs2910164 as a potential CRC risk factor are still unclear.^27,28^

 Thus, the present study aims to investigate, for the first time, the potential value of *miR-27a* (rs895819), *miR-146a* (rs2910164) and *miR-196a2* (rs11614913) variants with CRC development and its clinicopathological features in a Mexican population.

## Materials and Methods

###  Study Population

 This study was approved by the National Committee for Scientific Research of the Mexican Institute of Social Security (IMSS) (R-2019-785-171), following national and international ethical standards. Informed consent letters were signed by patients and control individuals, authorizing their participation and blood sample collection. This study included 369 individuals: 183 patients (108 men and 75 women) with histologically confirmed diagnosis of sporadic colorectal adenocarcinoma according to the International Guidelines for Colon and Rectal Cancer and the clinicopathological criteria of the medical staff of the Specialties Hospital of the Mexican Social Security Institute (IMSS) in Guadalajara, Mexico. All samples were collected consecutively from patients treated at this Center between 2019 and 2021.

 CRC stage was determined based on the TNM classification system. None of the included patients had undergone chemotherapy or radiotherapy at the time of sampling. The control group included 186 unrelated individuals (92 men and 94 women) with negative colonoscopy for malignancy and of the same age as the patient group. All individuals participating in this study were ethnically mestizos from the metropolitan area of Guadalajara, Mexico. The exclusion criteria for patients and controls included (1) Negative diagnosis of inflammatory bowel disease or autoimmune disease, (2) Negative history of hereditary cancer. Personal data including age, sex, drinking and smoking habits, background family members, and clinical and pathological features, were obtained from hospital records.

###  Genotyping

 Standard protocols were followed to extract genomic DNA from blood samples (Miller, Dykes, and Polesky 1988). Identification of genotypes for the variants rs895819 (A > G) of *miR-27a*, rs11614913 (T > G) of *miR-196a*2 and rs2910164 (C > G) of *miR-146a* was accomplished by PCR-RFLP. For all three variants (rs895819, rs11614913 and rs2910164), PCR-RFLPs reactions were performed using the primers and procedure described by Parchami et al^9^ and Min et al.^27^ Around 10% of the samples, chosen randomly, were re-genotyped and the findings were completely (100%) consistent.

###  Statistical Analysis

 The allelic and genotypic frequencies of the variants in both groups were estimated by direct counting. Hardy-Weinberg equilibrium (HWE) was evaluated by chi-square. In order to establish the association of genotypes and alleles with CRC and with the demographic and clinicopathological features, odds ratios (ORs) and confidence intervals (CIs) were calculated using SPSS v.28.0 (SPSS Inc., Chicago, IL, USA). Subsequently, a multiple logistic regression analysis was performed with the SPSS software, considering potential confounding factors such as sex, age, smoking and alcohol consumption. *P* values < 0.05 were considered statistically significant for all statistical analyses. Bonferroni correction was applied to adjust *P* values (*P* < 0.016).

## Results

###  Demographic and Clinical Features of the Study Groups

 The study consisted of 183 people diagnosed with CRC (108 men and 75 women), and a control group of 186 healthy participants (92 males and 94 females), all of whom were efficiently genotyped for the variants *miR-27a* (rs895819 A > G), *miR-196a2* (rs11614913 T > C) and *miR-146a* (rs2910164 C > G) variants. Demographic, clinical and anatomopathological data of CRC patients and control subjects included in the study are shown in Table 1. The mean age of the CRC group was 58.31 years (range 20 to 92 years), while the control group had a mean age of 59.88 years (40 to 91 years). There were significant differences between the two groups with regard to smoking and drinking for patients and controls (*P* = 0.001). Regarding the clinical and pathological characteristics of CRC patients, 67% had stage III-IV tumors, 29% had metastases, and 64% had tumors located in the rectum.

**Table 1:** Clinical Features of Study Subjects

**Features**	**CRC Group** **n=183 (100%)**	**Control Group** **n=186 (100%)**	***P *****Value**
Mean Age (years SD)	58.31 ( ± 12.45)	59.88 ( ± 12.74)	0.232
Age (y)			
< 50	34 (19)	50 (27)	0.075
> 50	149 (81)	136 (73)	
Gender			
Female	75 (41)	94 (51)	0.082
Male	108 (59)	92 (49)	
Smoking status			
Yes	61 (33)	28 (15)	**0.001**
No	122 (67)	158 (85)	
Drinking status			
Yes	54 (30)	22 (12)	**0.001**
No	129 (70)	164 (78)	
Clinical stage TNM			
I	4 (2)		
II	57 (31)		
III	69 (38)		
IV	53 (29)		
Tumor location			
Colon	66 (36)		
Rectum	117 (64)		
Site of metastasis			
Liver	18 (10)		
Lung	7 (4)		
Liver and lung	9 (5)		
Peritoneum	2 (1.17)		
Lung and peritoneum	2 (1.17)		
Ovary	2 (1.17)		
Brain	1 (0.49)		
NA	12 (7)		
Treatment response			
Non response	50 (27.22)		
Partial response	50 (27.22)		
Complete response	82 (45)		
NA	1 (0.56)		

*P* values were calculated by the chi-square test. Bold values indicate statistically significant findings. SD, standard deviation; NA, not available.

###  miR-27a, miR-196a2 and miR-146a Variants in the Study Groups 

 The *miR-27a *and *miR-196a2* variants in CRC patients and control individuals showed significant differences (Table 2). In the control group, the three analyzed SNPs were in HWE (*P* > 0.05). For the rs895819 variant, the HWE was X_2_ = 0.7878 (*P* = 0.37); for the rs11614913 variant, it was X_2_ = 0.1.3773 (*P* = 0.24) and for the rs2910164 variant, X_2_ = 0.5832 (*P* = 0.44). The genotype G/G of the *miR-27a* rs895819 A > G variant was found in 19% (35/183) of the CRC patients and in 12% (22/186) of the controls; this difference was statistically significant (OR = 2.29; 95% CI = 1.22–4.28, *P* = 0.013). Under a dominant pattern of allelic interaction (A/G + G/G vs. A/A), the G allele was found to be associated with CRC (OR = 1.79; 95% CI = 1.17-2.73, *P* = 0.008). Allelic frequencies were also significantly different; G allele carriers had an increased susceptibility for CRC (OR = 1.57; 95% CI = 1.16-2.12, *P* = 0.003).

**Table 2:** Genotypes and Allelic Frequencies of the miRNAs Analyzed in Study Subjects

**Genotype**	**CRC Group** **n=183 (%)**	**Control Group** **n=186 (%)**	**OR (95% CI)**	***P***** Value**
***miR-27a*** **(rs895819)**				
A/A	61 (33)	88 (47)	1.00 (Reference)	
A/G	87 (48)	76 (41)	1.65 (1.05- 2.58)	0.037
G/G	35 (19)	22 (12)	2.29 (1.22- 4.28)	**0.013**
A/G + G/G vs. A/A	122 (62)	98 (53)	1.79 (1.17 - 2.73)	**0.008**
Allele				
A	209 (57)	252 (69)	1.00 (Reference)	
G	157 (43)	120 (31)	1.57 (1.16 – 2.12)	**0.003**
***miR-196a2*** **(rs11614913)**				
T/T	25(14)	43 (23)	1.00 (Reference)	
T/C	99(54)	101(54)	1.68 (0.95 – 2.96)	0.031
C/C	59 (32)	42(22)	2.41 (1.28 – 4.54)	**0.009**
T/C + C/C vs. T/T	158(68)	143 (77)	1.90 (1.10 – 3.26)	0.027
Allele				
T	149 (41)	185 (49)	1.00 (Reference)	
C	217 (59)	187 (51)	1.44 (1.07 – 1.92)	**0.016**
***miR-146a*** **(rs2910164)**				
C/C	17 (11)	20 (11)	1.00 (Reference)	
C/G	82 (44)	75 (40)	1.09 (0.54 - 2.18)	0.940
G/G	84(45)	91 (49)	0.92 (0.46 - 1.83)	0.957
C/G + G/G vs. C/C	166 (91)	166 (89)	1.00 (0.51 - 1.92)	1.000
Allele				
C	116 (32)	115 (31)	1.00 (Reference)	
G	250 (68)	257 (69)	0.94 (0.67 - 1.31)	0.793

Bonferroni test was used to adjust the *P* value (0.016). Bold values indicate statistically significant findings.

 Regarding the *miR-196a2* rs11614913 T > C variant, the C/C genotype exhibited significant differences (OR = 2.41; 95% CI = 1.28-4.54, *P*= 0.009) between the patients and the control group. Allele frequency analysis also indicated a borderline association between the C allele and increased risk of CRC (OR = 1.44, 95% CI = 1.07-1.92, *P* = 0.016).

 Regarding the rs2910164 C > G variant of *miR-146a*, we found no statistical significance between the groups.

###  Analysis of miR-27a, miR-196a2 and miR-146a Genotypes by Clinicopathological Features

 The relationships of the genotypes *miR-27a* rs895819, *miR-196a2* rs11614913 and *miR-146a* rs2910164 with age, sex, tumor location and TNM stage are shown in Tables 3, 4 and 5. Women carrying the *miR-27a* rs895819 variant showed a significantly increased risk of CRC in the presence of the G/G genotype (OR = 4.76, 95% CI = 1.63-13.86, *P* = 0.006); with the dominant AG + GG vs A/A model, women also showed an increased susceptibility to development of CRC (OR = 2.37, 95% CI = 1.26-4.44, *P* = 0.010). Patients younger than 50 years, carrying genotypes A/G and G/G, showed higher susceptibility to CRC (OR = 4.14, 95% CI = 1.42-12.01, *P* = 0.014, and OR = 10.35, 95% CI = 2.49-42.99, *P* = 0.001, respectively). Analysis of disease development showed that patients carrying the A/G genotype were associated with early TNM stages (I + II) (OR = 2.46, 95% CI = 1.26-4.80, *P* = 0.011). Analysis by tumor location in the colon showed statistical significance under the dominant allelic interaction pattern (A/G + G/G vs. A/A) (OR = 3.19; 95% CI = 1.75-5.79, *P* = 0.001).

**Table 3:** Association of the miR-27a rs895819 Variant with Clinicopathological Variables

**miR-27a (rs895819)**						
**Variable **	**CRC/Control**			**OR (95% CI); *****P***** Value**		
	**AA**	**AG**	**GG**	**AG vs. AA**	**GG vs. AA**	**AG+GG vs. AA**
Gender						
Male	36/37	51/39	21/16	1.34 (0.72 - 2.49); 0.436	1.34 (0.60 – 2.99); 0.591	1.34 (0.75 - 2.39); 0.389
Female	25/51	36/37	14/6	1.98 (1.02 - 3.85); 0.061	**4.76 (1.63 – 13.86); 0.006**	**2.37 (1.26 – 4.44); 0.010**
Age (y)						
< 50	7/29	17/17	10/4	**4.14 (1.42 – 12.01); 0.014**	**10.35 (2.49 – 42.99); 0.001**	**5.32 (1.95– 14.52); 0.001**
> 50	54/59	70/59	25/18	1.29 (0.78 - 2.15); 0.380	1.51 (0.74 – 3.08); 0.328	1.34 (0.83 – 2.16); 0.267
TNM stage						
I + II	16/88	34/76	11/22	**2.46 (1.26 - 4.80); 0.011**	2.75 (1.11 – 6.75); 0.044	**2.52 (1.33 – 4.78); 0.006**
III + IV	45/88	53/76	24/22	1.36 (0.82 - 2.25); 0.278	2.13 (1.07 – 4.21); 0.042	1.53 (0.96 – 2.45); 0.091
Tumor location						
Colon	18/88	57/76	07/22	**3.66 (1.98 – 6.76); 0.000**	1.55 (0.57 - 4.18); 0.542	**3.19 (1.75 – 5.79); 0.001**
Rectum	42/88	39/76	11/22	1.07 (0.63 – 1.83); 0.896	1.04 (0.46 – 2.35); 1.000	1.06 (0.64 – 1.76); 0.894

Bonferroni test was used to adjust the *P* value (0.016). Bold values indicate statistically significant findings.

**Table 4:** Association of the *miR-196a2* rs11614913 Variant with Clinicopathological Variables.

***miR-196a2*** ** rs11614913**						
**Variable **	**CRC/Control**			**OR (95% CI); *****P *****Value**		
	**TT**	**TC**	**CC**	**TC vs. CC**	**CC vs. TT**	**TC+CC vs. TT**
Gender						
Male	13/19	60/48	35/25	1.82 (0.82 – 4.07) 0.066	2.04 (0.85 – 4.89); 0.161	1.90 (0.88 – 4.10); 0143
Female	12/23	39/53	24/18	1.41 (0.62 – 3.17); 0.176	2.55 (1.01 – 6.46); 0.025	1.7 (0.78 – 3.69); 0.082
Age (y)						
< 50	4/11	19/29	11/10	1.80 (0.49 – 6.49) 0.182	3.02 (0.72 – 12.63); 0.076	2.11 (0.61 – 7.30) 0.120
> 50	21/31	80/72	48/33	1.64 (0.86 – 3.10) 0.057	2.14 (1.05 – 4.36) 0.017	1.79 (0.97 – 3.31) 0.026
TNM stage						
I + II	8/43	39/101	14/42	2.07 (0.89 – 4.80); 0.041	1.79 (0.68 – 4.71); 0.113	2.02 (0.89 – 4.59); 0.041
III + IV	17/43	60/101	45/42	1.50 (0.78 – 2.86); 0.093	**2.71 (1.34 – 5.52); 0.007**	1.50 (0.82 – 2.75); 0.079
Tumor location						
Colon	12/43	32/101	22/42	1.13 (0.53 – 2.41); 0.295	1.87 (0.82 – 4.26); 0.063	1.35 (0.66 – 2.75); 0.169
Rectum	14/43	66/101	37/42	2.00 (1.01 – 3.95); 0.020	**2.70 (1.28 – 5.71); 0.013**	**2.21 (1.15 – 4.25); 0.007**

Bonferroni test was used to adjust the *P* value (0.016). Bold values indicate statistically significant findings.

**Table 5:** Association of the *miR-146a* rs2910164 Variant with Clinicopathological Variables

***miR-146a*** ** rs2910164**						
**Variable **	**CRC/Control**			**OR (95% CI); *****P *****value**		
	**CC**	**CG**	**GG**	**CG vs. CC**	**GG vs. CC**	**CG +GG vs CC**
Gender						
Male	7/10	54/42	44/42	1.83 (0.64 – 5.23); 0.376	1.49 (0.52 – 4.29); 0.626	1.66 (0.60 – 4.57); 0.455
Female	7/7	28/33	40/49	0.84 (0.26 – 2.71); 1.000	0.83 (0.26 – 2.68); 0.977	0.83 (0.28 – 2.51); 0.973
Age (y)						
< 50	3/7	17/21	14/22	1.88 (0.42– 8.43); 0.772	1.48 (0.32 – 6.71); 0.884	1.68 (0.40 – 7.02); 0.707
> 50	14/13	65/54	70/69	1.11 (0.48 – 2.58); 0.962	0.94 (0.41 – 2.14); 0.887	0.01 (0.46 – 2.25); 1.000
TNM stage						
I + II	5/17	29/75	27/91	1.31 (0.44 – 4.30); 0.817	1.00 (0.34 - 2.98); 1.000	1.14 (0.40 - 3.25); 1.000
III + IV	12/17	53/75	57/91	1.00 (0.44 – 2.26); 1.000	0.88 (0.39 – 1.99); 0.935	0.93 (0.43 – 2.04); 1.000
Tumor location						
Colon	9/17	29/75	28/91	0.76 (0.29 - 1.97); 0.760	0.65 (0.25 – 1.67); 0.525	0.70 (0.28 – 1.72); 0.595
Rectum	8/17	53/75	56/91	1.50 (0.60 - 3.73); 0.512	1.30 (0.52 – 3.22); 0.719	1.39 (0.58 – 3.34); 0.592

Bonferroni test was used to adjust the *P *value (0.016). Bold values indicate statistically significant findings.

 Regarding the rs11614913 variant of *miR-196a2*, CRC patients showed a significant increased risk in the presence of the C/C genotype (OR = 2.41; 95% CI = 1.28-4.54, *P* = 0.009). We also observed a significant association of advanced TNM stages (III + IV) with the presence of the C/C genotype (OR = 2.71, 95% CI = 1.34-5.52, *P* = 0.007) and an increased risk was observed for tumor location in the rectum in patients with the presence of C/C genotype.

 For *miR-146a* rs2910164, we found no statistical association when comparing by sex, age, TNM stage and tumor location in the groups.

###  Multivariable Logistic Regression Analysis with Confounding Variables

 The results of the multiple logistic regression analysis are shown in Table 6 for confounding variables. Tobacco and alcohol use showed a statistical significance in the presence of the three variants analyzed (OR = 2.224; 95% CI = 1.281-3.861; *P* = 0.005 and OR = 2.668; 95% CI = 1.463-4.868; *P* = 0.001, respectively), suggesting that these variables increased the risk of developing CRC.

**Table 6:** Logistic Regression Analysis for the miRNAs Analyzed with Confounding Variables

**Independent Variable**	**Regression Coefficient**	**Standard Error**	**Wald Test**	**Degrees of Freedom**	***P *****Value**	**OR (95% IC)**
Age > 50 vs. < 50	0.482	0.271	3.166	1	0.075	1.620 (0.952-2.755)
Sex Male vs. Female	0.191	0.225	0.717	1	0.397	1.210 (0.778-1.882)
Smoking status Yes vs. No	0.799	0.281	8.069	1	**0.005**	**2.224 (1.281-3.861)**
Drinking status Yes vs. No	0.981	0.307	10.237	1	**0.001**	**2.668 (1.463-4.868)**
rs895819 AG + GG	0.668	0.230	8.401	1	**0.004**	**1.950 (1.241-3.063)**
rs11614913 TC + CC	0.894	0.301	8.810	1	**0.003**	**2.445 (1.355-4.411)**
rs2910164 CG + GG	0.048	0.370	0.017	1	0.896	1.050 (0.508-2.167)
Constant	-2.013	0.353	32.512	1	**0.000**	
Model	X^2^ = 47.943 d.f. = 7. *P* = **0.000**					

Bonferroni test was used to adjust the *P* value (0.016); Bold values indicate statistically significant findings.

## Discussion

 According to the multifactorial and polygenic model of colorectal carcinogenesis, various combinations of genetic variations in susceptibility genes with low penetrance contribute to increased CRC risk. Several studies have shown that the expression profiles of miRNAs are different between normal and tumoral tissues in CRC patients.^8^ It is now known that miRNAs are involved in the silencing of several genes implicated in CRC.^5^ Recently, a new class of functional variants in miRNAs or their binding sites has attracted great interest. Three SNPs: *miR-27a* rs895819 A > G, *miR-196a2* rs11614913 T > C and *miR-146a* rs2910164 C > G have been studied in different populations regarding their susceptibility to development of cancer, with rather controversial results.^12,13,16,26^

 The results of this study carried out in the Mexican population show an evident increase of CRC in people older than 50 years (81%); a finding that has been previously reported in other studies^5^ and agrees with the statistics reported worldwide.^29^ Regarding alcohol and tobacco consumption, significant differences were observed between the groups, similar to the results described by Tsong et al in 2007, who reported that alcohol and tobacco consumption play an important role in colorectal carcinogenesis in the Chinese population.^30^

 In this study, the risk of developing CRC was statistically evident in individuals carrying the G/G genotype for the *miR-27a* variants (rs895819 A > G), and the C/C genotype for the *miR-196a2* variant (rs11614913 T > C). Similar findings were reported in previous studies performed in patients with different types of cancer, including CRC.^6,12,31^

 Regarding the rs895819 variant of *miR-27a*, the G/G genotype was found to be significantly associated with CRC risk, suggesting it to be a genetic factor for CRC susceptibility. Furthermore, we found similar association in female patients < 50 years, with early TNM stage (I + II) and tumor location in the colon. These results are similar to reports by Cao et al.^6^ Some studies have shown overexpression of *miR-27a* in colorectal cell lines such as HT29, SW480 and RKO.^32^ It has been also described that rs895819, which is located in the terminal loop of the pre-miRNA, might affect the second structure of mature *miR-27a*, leading to abnormal function.^13^ Relatively higher expression of miR-27a has been observed in CRC tissues from patients carrying the G/G genotype or the G allele (AG/GG) compared to the A/A genotype.^6^ Moreover, it has been suggested that miR-27a functions as an oncogene in CRC.^33^ Tian & Bian demonstrated that increased expression of miR-27a leads to inhibition of PLK2 and ZBTB10 protein expression, resulting in increased colony formation and cell viability, as well as suppression of late apoptosis.^34^ These results could explain the elevated risk of CRC associated with the G/G genotype of rs895819.^35^ Another possibility is that the rs895819 polymorphism may down-regulate the miR-27a expression, which would affect the tumor suppressor function of miR-27a. From this perspective, the variant rs895819 may promote the occurrence and development of CRC by attenuating the tumor suppressor effect of miR-27a.^36^ Consequently, rs895819 might be acting as a predisposing factor for CRC, which corroborates the findings of our investigation.

 Regarding the rs11614913 T > C variant of *miR-196a2*, a significant difference was observed between CRC patients and controls for the C/C genotype. In this study, the C allele was marginally associated with increased susceptibility. In addition, a significant association was found between higher TNM stages (III + IV) and tumor location in the rectum. These findings are similar with reports from other populations.^31^

 In CRC, a positive correlation has been observed between increased migration, invasiveness and increased expression levels of *miR-196a2.*^17^ The mechanism data suggest that irregular expression of mature *miR-196a2 *may alter cell viability and migration ability, which may eventually increase susceptibility to ovarian cancer.^37^ Such results suggest that the rs11614913 polymorphism affects the conversion of pre-miRNA to its mature form. On the other hand, it is conceivable to use the miR-196 family as potent inhibitors of metastasis by binding to the *HOXC8* transcription factor.^3^ The study by Song showed that *in vivo* and *in vitro*, the C allele of miR-196a2 (rs11614913) induced expression of mature miR-196a2.^37^

 Another study in patients with gastrointestinal cancer reported that *miR-196a2* represses three genes (*ANXA1*, *DFFA*, and *PDCD4*) that actively participate in apoptosis; however, it was remarked that in patients with CRC, deregulation of *miR-196a2 *and* ANXA1 *is associated with poor prognosis (advanced stages, high pathological grade, larger tumor size).^4^

 Protein interaction analysis of the *miR-196a2* target proteins shows many proteins involved in particular biological processes such as cell cycle regulation, cell signaling pathways, chromatin condensation, DNA repair, activation of apoptosis through its binding to CASP3, among other processes that, when altered, can favor the development of CRC.^4^ No significant differences were observed for the rs2910164 variant of *miR-146a *in this study. Consistent with the results obtained for the rs2910164 variant of *miR-146a* in the Chinese population, the presence of this variant was not associated with increased susceptibility to CRC.^38^

 Finally, the multivariable analysis showed, for the first time, that tobacco and alcohol consumption are a risk factor for CRC in carriers of the A/G or G/G genotypes of the rs895819 variant, and T/C or C/C genotypes of the rs11614913 variant. Potentially modifiable lifestyle factors, such as alcohol and cigarette consumption, have been associated with CRC risk in multiple studies conducted in Western populations.^39,40^

 In conclusion, the results of the present study indicate that the G/G genotypes of rs895819 (*miR-27a*) and C/C of rs11614913 (*miR-196a2*) are significantly associated with colorectal carcinogenesis. Functional studies including larger sample sizes should be performed to corroborate the results of this study.
